# Centered on Breast Cancer

**DOI:** 10.1289/ehp.115-a132

**Published:** 2007-03

**Authors:** Luz Claudio

A relationship between early menarche in girls and later development of breast cancer has long been observed. Some environmental factors, such as diet and exposure to endocrine disruptors and other chemicals, could affect children’s timing and pace of puberty and development. This leads to the question of whether exposure to these agents may also lead to breast cancer later in life. That is the scientific premise for the establishment of the Breast Cancer and the Environment Research Centers (BCERCs), which presented results of ongoing studies at their third annual meeting on 2–3 November 2006.

The centers are co-funded by the NIEHS and the National Cancer Institute (NCI) Epidemiology and Genetics Research Program. They work together to integrate community outreach and two lines of research: 1) basic biology of the mammary gland and its development using animal models, and 2) epidemiological studies of how environmental factors affect puberty in girls. Four centers were established across the nation: the Fox Chase Cancer Center in Philadelphia (collaborating with Mount Sinai School of Medicine in New York and the University of Alabama at Birmingham), the University of Cincinnati in Ohio (collaborating with Cincinnati Children’s Hospital Medical Center), the University of California, San Francisco (collaborating with numerous partners including Lawrence Berkeley National Laboratory), and Michigan State University in East Lansing.

“Using a transdisciplinary scientific approach, the centers are able to investigate possible windows of susceptibility during pubertal development more comprehensively and on a larger scale,” said Shannon Lynch, a program analyst at the NCI. The BCERCs’ ability to work closely together, share resources, compare findings, and establish common research protocols is especially important in epidemiological studies. With each center recruiting a number of girls from diverse racial/ ethnic, geographical, and socioeconomic backgrounds, the data that are ultimately generated should be more robust due to a greater number of samples that are more representative of different populations.

## Research Update

One example of the kind of multidisciplinary data that can be generated by this collaboration can be found in a paper presented at the meeting, which was later published in the January 2007 issue of *EHP*. Center researchers collaborated with the CDC to analyze urine samples collected from 6- to 8-year-old girls to assess the levels of bio-markers of exposure to a number of phytoestrogens, phthalates, and phenols. Many of these compounds are known or suspected to cause endocrine disruption.

The authors found that these potentially hormonally active compounds were widely detectable in the girls studied. For several of the compounds, concentrations varied by geographic location, body size, or race/ethnicity such that meaningful comparisons may become possible. It is still too early to tell whether exposure to these compounds will be associated with puberty, but so far the preliminary data show that exposures can be substantial and perhaps biologically relevant.

Each center is also producing new research results within its own populations of study. The University of California center, for example, working together with the Kaiser Permanente Division of Research, has recruited 444 girls, along with their families, to study how environmental exposures may affect growth and development. The study participants are members of Kaiser Permanente of Northern California, an integrated prepaid health care system, so researchers have access to early medical information for the girls including birthweight.

In preliminary analyses, Larry Kushi, associate director of the Kaiser Permanente Division of Research, and colleagues found that higher birthweight predicted risk of overweight in 6- to 7-year-old girls. It is important to assess peripubertal obesity in girls enrolled in the study because obesity is a strong predictor of breast development; how obesity may affect timing and degree of puberty development is one of the subjects of this investigation. A significant proportion (29%) of girls enrolled in this study were classified as overweight, defined as being at or above the 85th percentile for body mass index. Ongoing analyses are assessing the levels of pubertal development in the cohort. The preliminary findings suggest that the black and Latina girls are having a higher prevalence of early puberty onset.

Investigators at the meeting also presented results of studies conducted in the basic science components of their centers. Deborah J. Clegg, an assistant professor in the Department of Psychiatry at the University of Cincinnati College of Medicine, conducted a yet-unpublished study to assess how caloric content and type of dietary fat eaten may affect obesity and carcinogenesis susceptibility in laboratory rats. Her team tested high- and low-fat diets rich in olive oil, fish oil, safflower oil, or butter (a “1950s diet”). They found that the high-fat olive oil diet accelerated puberty and increased propensity to carcinogenesis in the exposed rats. Additionally, they found that the high-fat diets enriched with safflower oil and fish oil increased body weight and susceptibility to carcinogenesis. Interestingly, however, the high-fat butter diet did not increase body weight or carcinogenicity.

## Defining Messages

In addition to the basic and epidemiological research being conducted, each center also has a Community Outreach and Translation Core (COTC). The COTCs have different functions within each center, including sharing information with the community, conducting research on translating science findings, and studying recruitment and retention strategies.

The COTC at Michigan State University presented data on the types of meaningful messages about breast cancer that women remember for long periods of time and the sources of such messages. Based on responses from 137 women who completed an online questionnaire, researchers concluded that most messages were recalled from the mass media. Most messages were about the detection of breast cancer rather than its prevention. These findings demonstrate that the media has a strong influence on the messages received by women, perhaps more influence than the medical community.

The issue of what health messages can impact the community at large resonated with many participants present at the meeting, which included leaders of the breast cancer control advocacy community, who are active participants in the centers. Beth Hartung, a member of the Young Survival Coalition (a network of breast cancer survivors who are 40 or younger), expressed this succinctly as she asked a panel of center scientists, “What do we tell our daughters [about their risk of breast cancer]?” Program administrator Les Reinlib of the NIEHS Division of Extramural Research and Training, himself the father of two daughters and husband to a breast cancer survivor, answered, “We tell them to make lifestyle choices that improve general health.”

For now, until more definitive research on specific environmental exposures has been completed, exercise and good nutrition are the two main lifestyle choices that can help in the prevention of disease. And despite the uncertainty that remains, there have been many scientific advances that have extended the lives of women diagnosed with breast cancer.

Andrea Ice, cofounder of the Sisters Network (a national breast cancer survivorship organization for black women), has seen this first-hand. “Since my initial diagnosis in 1989, the biggest change has been that women see other women who have survived the disease for many years,” she says. “It is no longer a death sentence.” The hope is that the BCERCs will be able to add prevention to the progress made in breast cancer research.

## Figures and Tables

**Figure:**
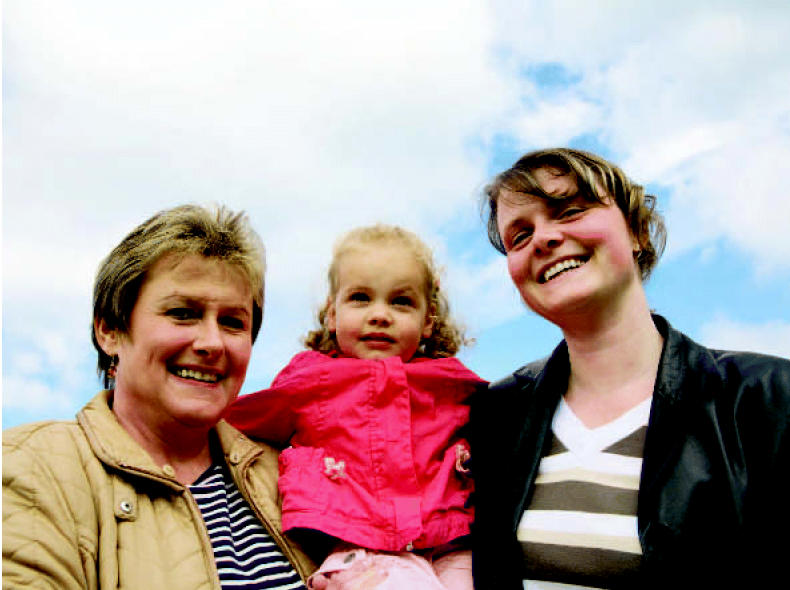
Window to the future The Breast Cancer and the Environment Research Centers are looking at ways that early environmental exposures may influence later breast cancer risk.

